# Soil Metatranscriptomes Under Long-Term Experimental Warming and Drying: Fungi Allocate Resources to Cell Metabolic Maintenance Rather Than Decay

**DOI:** 10.3389/fmicb.2019.01914

**Published:** 2019-08-20

**Authors:** Adriana L. Romero-Olivares, Germán Meléndrez-Carballo, Asunción Lago-Lestón, Kathleen K. Treseder

**Affiliations:** ^1^Department of Ecology and Evolutionary Biology, University of California, Irvine, Irvine, CA, United States; ^2^Department of Electronics and Telecommunications, Ensenada Center for Scientific Research and Higher Education, Ensenada, Mexico; ^3^Department of Medical Innovation, Ensenada Center for Scientific Research and Higher Education, Ensenada, Mexico

**Keywords:** metatranscriptome, tradeoff, fungi, soil carbon, decomposition, global warming, CAZy, COG

## Abstract

Earth’s temperature is rising, and with this increase, fungal communities are responding and affecting soil carbon processes. At a long-term soil-warming experiment in a boreal forest in interior Alaska, warming and warming-associated drying alters the function of microbes, and thus, decomposition of carbon. But what genetic mechanisms and resource allocation strategies are behind these community shifts and soil carbon changes? Here, we evaluate fungal resource allocation efforts under long-term experimental warming (including associated drying) using soil metatranscriptomics. We profiled resource allocation efforts toward decomposition and cell metabolic maintenance, and we characterized community composition. We found that under the warming treatment, fungi allocate resources to cell metabolic maintenance at the expense of allocating resources to decomposition. In addition, we found that fungal orders that house taxa with stress-tolerant traits were more abundant under the warmed treatment compared to control conditions. Our results suggest that the warming treatment elicits an ecological tradeoff in resource allocation in the fungal communities, with potential to change ecosystem-scale carbon dynamics. Fungi preferentially invest in mechanisms that will ensure survival under warming and drying, such as cell metabolic maintenance, rather than in decomposition. Through metatranscriptomes, we provide mechanistic insight behind the response of fungi to climate change and consequences to soil carbon processes.

## Introduction

Genetic mechanisms and strategies behind fungal resource allocation in response to warming remain unclear despite being critical to understand ecosystem-scale carbon (C) dynamics under global climate change. For example, warming-induced soil carbon losses in high-latitude ecosystems are predicted to provide a positive feedback to global warming (Allison and Treseder, 2011). However, litter mass loss and microbial CO_2_ emissions seem to decrease after a decade or more exposed to warming (Melillo et al., 2017; Romero-Olivares et al., 2017a, b). Simultaneously, warming changes the community composition of microbes, and these changes have the potential to affect ecosystem-scale C losses (e.g., Pold et al., 2015; Treseder et al., 2016; Morrison et al., 2019). How do these community changes and ecosystem-scale C losses feedback to global warming? An intimate look into the energetic investment of fungi under warming can help elucidate priorities on resource allocation under global climate change and facilitate predictions on the fate of soil C.

Microbes are mediators of biogeochemical cycles in soil. In particular, fungi are decomposition power-houses (Berlemont, 2017). In high-latitude ecosystems, fungi are the major decomposers of forest litter (Baldrian, 2017). Previous studies in high-latitudes reported that warming treatments significantly alter the fungal community, favoring soil saprotrophs and ectomycorrhizal fungi, while disfavoring yeasts (Treseder et al., 2016; Solly et al., 2017). In this biome, warming can also cause soil drying, owing to higher evapotranspiration rates (Allison and Treseder, 2011). Soil drying can contribute to these shifts in fungal communities under warming treatments (Allison and Treseder, 2008; Treseder et al., 2016). But to understand how fungi respond to warming and drying, and to better predict ecosystem-scale C dynamics under global climate change, we must explore beyond community composition (Crowther et al., 2014).

Laboratory studies on the physiological responses of the model fungus *Neurospora discreta* show that under warming, *N. discreta* adapts its physiology and invests energy differently to increase its chances of survival (Romero-Olivares et al., 2015). More specifically, after 1500 generations exposed to warm temperature and a labile C substrate, *N. discreta* invested more energy toward reproduction rather than growth and decreased its carbon use efficiency (CUE) (Romero-Olivares et al., 2015). But when exposed to warm temperature, and either a labile or recalcitrant C substrate, *N. discreta* was less sensitive to temperature shifts and most sensitive to C substrates (Allison et al., 2018a). Moreover, *N. discreta* isolated from different parts of the world—and presumably adapted to different mean annual temperatures—did not differ in their response to temperature (Allison et al., 2018b). Altogether, these studies expose the complexity of evolutionary forces in soil and the different responses fungi may have. Temperature, as well as warming-induced biotic and abiotic environmental changes (e.g., warming-induced drying), may be strong evolutionary forces (Bennett and Lenski, 2007; Allison et al., 2018a).

Studies on the metabolic changes of fungi under environmental stress, warming included, have focused on short-term responses (i.e., 30–60 min) (e.g., Gasch et al., 2000; Albright et al., 2018). However, in ecosystem ecology, “stress” is considered a chronic condition that extends for long periods of time with physiological expenses for microbes (Schimel et al., 2007). These expenses are usually associated with reallocation of resources to ensure survival (i.e., tradeoffs) (Bennett and Lenski, 2007). The rate-yield tradeoff states that “extra energy devoted to resource acquisition speeds metabolic rate but reduces the net yield of energy” (Frank, 2010). Indeed, in microbes, CUE can decline with warming, because respiration maintenance processes are more temperature sensitive than growth processes (Farmer and Jones, 1976; Mainzer and Hempfling, 1976; Hall and Cotner, 2007; Allison, 2014; Rodríguez-Verdugo et al., 2014). On the long-term, species that consistently invest in cell metabolic maintenance mechanisms may be selected for under stressful conditions brought by global warming.

Certain fungal traits associated with stress-tolerance may be favorable under warming and drying. For example, the presence of melanin is known to be associated with drought-tolerance and is often found in fungi that inhabit ecosystems exposed to high-radiation, such as deserts (Harutyunyan et al., 2008; Belozerskaya et al., 2010; Azua-Bustos et al., 2012; Fernandez and Koide, 2013; Casadevall et al., 2017) Similarly, trehalose protects fungal cell membranes from desiccation, freezing, and heat shock (Treseder and Lennon, 2015). This trait is commonly found in arctic fungi (e.g., Gunde-Cimerman et al., 2003) where water availability is low, as well as in lichenicolous fungi which are known to be xerotolerant (Mittermeier et al., 2015). Under warming and drying, fungal taxa with stress-tolerance traits may thrive over those without those traits.

Exploring the fungal community composition and its traits, paired with physiological and metabolic profiles, can provide an integrative overview of the response of the fungal community to warming and drying. In addition, it offers insight into fungal adaptation strategies in the wake of climate change, and facilitates predictions on the fate of soil C. In this study, we used metatranscriptomes to get a mechanistic understanding of the allocation of resources in fungal communities exposed to a long-term warming treatment (which includes drying). We tested the following hypotheses:

### Cell Metabolic Maintenance Hypothesis

Under the warming treatment, fungi allocate more resources into cell metabolic maintenance as a strategy to ensure survival compared to fungi under control conditions.

### Decay Hypothesis

Under the warming treatment, fungi allocate less resources to decomposition compared to fungi under control conditions as a tradeoff for investing in cell metabolic maintenance.

### Stress-Tolerance Traits Hypothesis

Under the warming treatment, fungal taxonomical orders that house taxa with stress-tolerant traits are more abundant compared to control.

Altogether, we hypothesized that under the warming treatment, there is an ecological tradeoff that favors the allocation of resources toward cell maintenance and stress-tolerance over the allocation of resources toward decomposition. This tradeoff is driven by the increase in abundance of stress-tolerant fungal taxa in response to warming and associate drying.

## Materials and Methods

### Field Site

The study area is located in a mature black spruce (*Picea mariana*) forest in Delta Junction, Alaska, United States (63°55′N, 145°44′W) on the Fort Greely military base. Here, the vegetation is dominated by black spruce and an understory of lichens, shrubs, and mosses. The annual precipitation is approximately 303 mm^–1^ and a mean annual temperature of −2°C; the growing season starts around mid-May and ends in mid-September.

The warming experiment started in July 2005 as described in Allison and Treseder (2008). Greenhouses and neighboring control plots were established in pairs in a 1 km^2^ area. Control plots were left under ambient conditions while greenhouses warmed passively using closed-top chambers. The top plastic panels of the greenhouses were removed at the end of the growing season and re-installed at the beginning of the growing season the following year to allow snow fall to enter the warmed plots. Gutters and tubing were installed to minimize drying by directing precipitation into the greenhouses during the growing season. On average, the air inside the greenhouses was 1.6°C higher than in controls plots and the soil temperature at 5 cm depth was on average 0.5°C higher inside the greenhouses compared to control plots. The warming treatment reduced soil moisture by 22%—on average—due to higher evapotranspiration (Allison and Treseder, 2008).

### Sample Collection

We collected soil samples in the summer of 2015, after 10 years of the onset of warming. We used a 10 cm-tall corer to extract soil, homogenized 332 cm^3^ of soil inside a plastic sterile Whirl-Pak^®^, collected approximately one gram, and immediately soaked it in 5 ml of LifeGuard^TM^ Soil Preservation Solution (Qiagen, catalog 12868). Samples were kept in a cooler with ice for 24 h and then transferred to a −80°C freezer until processed 1 week later.

### Soil RNA Extraction and Sequencing

Samples were thawed on ice and centrifuged at 2500 × g for 5 min to remove LifeGuard^TM^ Soil Preservation Solution. We proceeded to extract RNA using RNA PowerSoil^®^ following the manufacturer’s instructions with modifications by Baldrian et al. (2012). Samples were cleaned using RNA clean & concentrator^TM^-25 kit (Zymo, catalog R1017) and DNAse treated with Turbo-DNA free^TM^ kit (Life Technologies, catalog AM1907). RNA was checked for quality on an Agilent 2100 Bioanalyzer at the University of California Irvine genomics high-throughput facility. Good quality samples were prepared for sequencing and sequenced at the Joint Genome Institute (JGI) (Nordberg et al., 2014). The JGI prepared paired-end libraries using Illumina RNAseq stranded library preparation kit following low and ultra-low input RNAseq with rRNA depletion protocols. Shortly, rRNA was removed from either 100 or 10 ng of RNA using Ribo-zero^TM^rRNA removal kit (Epicentre). Stranded cDNA libraries were generated using Illumina TruSeq^®^ RNAseq stranded kit. The rRNA depleted RNA was fragmented and reversed transcribed using random hexamers and SSII (Invitrogen) followed by a second strand synthesis. The fragmented cDNA was treated with end-pair, A-tailing, adapter ligation, and 10 or 15 cycles of PCR. Sequencing was carried out in a HiSeq 2500 system. Sequencing projects are deposited at the JGI with project ids: 1107-496, -499, -504, -507, -509, -514, -519, and -520.

### COG Cell Metabolic Maintenance Genes

For an overview of the investment of fungi in cell metabolic maintenance in control versus warmed plots, we analyzed metatranscriptomes using the JGI Integrated Microbial Genomes and Microbiomes (IMG/M) platform (Markowitz et al., 2005; Chen et al., 2016), specifically, we used the functional categories of Clusters of Orthologous Groups (COG) at 90% similarity with an *E* value threshold of 10^–5^. We chose COG categories because this classification provides information on the functional characteristics of a community of microbes by using a group of proteins found to be orthologous across lineages (Tatusov et al., 2000). We used the COG functional category of “metabolism” because it is the only category that includes proteins involved exclusively in cell metabolic maintenance, such as the transport and metabolism of amino acids, nucleotides, carbohydrates, coenzymes, lipids, and inorganic ions; energy production and conversion; and secondary metabolite biosynthesis.

### CAZy Genes

Metatranscriptomes were quality trimmed by removing adapters with Trimmomatic (v 0.35) using ILLUMINA TruSeq3-PE adapters with sliding window 4:15 and dropping reads below 25 bases long (Bolger et al., 2014). Quality control of trimmed samples was carried out with FastQC (v 0.11.2) (Andrews, 2010). Metatranscriptomes were filtered with sortmeRNA (v 2.1) to remove all rRNA sequences (Kopylova et al., 2012). Filtering was done against bacteria and archaea 16 s- and 23 s-, as well as eukaryote 18 s- and 28 s-, and 5 s- and 5.8 s-SILVA databases (Quast et al., 2013). The rRNA-free metatranscriptomes were used for downstream analyses.

Four metatranscriptomes from control plots and four metatranscriptomes from warmed plots were selected to assemble a *de novo* reference meta-transcriptome with Trinity (v 2.3.2) (Haas et al., 2013). We used bowtie2 (v 2.2.7) to map reads to our reference meta-transcriptome (Langmead and Salzberg, 2013) and Samtools (v 1.3) for sorting and indexing (Li et al., 2009). We used Trinity toolkit (i.e., Trinity transcript quantification) to quantify abundance of transcripts and ran differential expression analysis with edgeR (v 3.3.2) using a *p*-value cutoff of 0.01. We used the Carbohydrate Active Enzyme (CAZy) database (Lombard et al., 2014) and Transdecoder (v 2.0.1)^1^ to find coding regions within transcripts. We created an annotated database with Trinotate (v 3.0.0)^2^ and hmmer (v 3.0) (Bryant et al., 2017) with an *E* value threshold of 10^–5^. The CAZy database has six different classes based on structurally related functional domains of enzymes that degrade, create, or modify glycosidic bonds. We used classes involved in catabolism, and excluded classes involved in anabolism (i.e., glycosyl transferases). Briefly, auxiliary activities (AA) is a class that has ligninolytic enzymes and lytic polysaccharide monooxygenases involved in the breakdown of lignin. Carbohydrate-binding modules (CBM) has enzymes that assist in the decomposition of cellulose and other types of complex carbohydrates. Carbohydrate esterases (CE) includes enzymes involved in the degradation of hemicellulose and pectin. Glycoside hydrolases (GH) is a wide group of enzymes that hydrolyze glycosidic bonds between carbohydrates or other non-carbohydrate molecules. Finally, polysaccharide lyases (PL) participate in the decomposition of acidic polysaccharides such as starch and chitin (Lombard et al., 2014). The full pipeline and scripts were deposited at https://github.com/adriluromero/warming_AK.

### Taxonomical Composition Analysis

We assessed fungal community composition in control and warmed plots using metatranscriptomes and M5NR database within MG-RAST (Meyer et al., 2008). Metatranscriptomes were deposited in the MG-RAST metagenomics analysis server with the following ID: mgm4761695.3, mgm4762031.3, mgm4762032.3, mgm4762033.3, mgm4762030.3, mgm4762034.3, mgm4762035.3, mgm4762036.3. We filtered results using an *E* value threshold of 10^–5^ and kept only hits for fungi. We used data at the order level because previous work by Treseder et al. (2016) showed that variance in warming treatment response was greatest at this level. In addition (Randle-Boggis et al., 2016) recommends focusing on order when using M5NR in MG-RAST, because this taxonomic level yields that highest numbers of correctly annotated sequences.

### Statistical Test

For all statistical tests we used R (R Core Team, 2018) with ranked data, since the unranked data did not meet assumptions of normality. We performed a linear mixed-effects model using the function lmer from the R package lme4 (Bates et al., 2015). In our model, plot was random effect, treatment was fixed effect, and transcription (i.e., gene counts for COG and CAZy) or relative abundance (for taxonomical order) was the dependent variable. COG metabolic maintenance subcategories, CAZy categories, or taxonomical order were categorical variables. We performed a Type III ANOVA in our model, to test for interactions; we used Satterthwaite’s method within lmerTest package (Kuznetsova et al., 2017) in R. This method provides *p*-values for the *F* values produced with the lmer function. We used *post hoc t*-test to estimate significant differences in gene counts of each categorical variable between control and warmed plots. Since we ranked the data, all statistical tests were non-parametric. All statistical scripts and test results are deposited at github.com/adriluromero/warming_AK.

## Results

**FIGURE 1:**
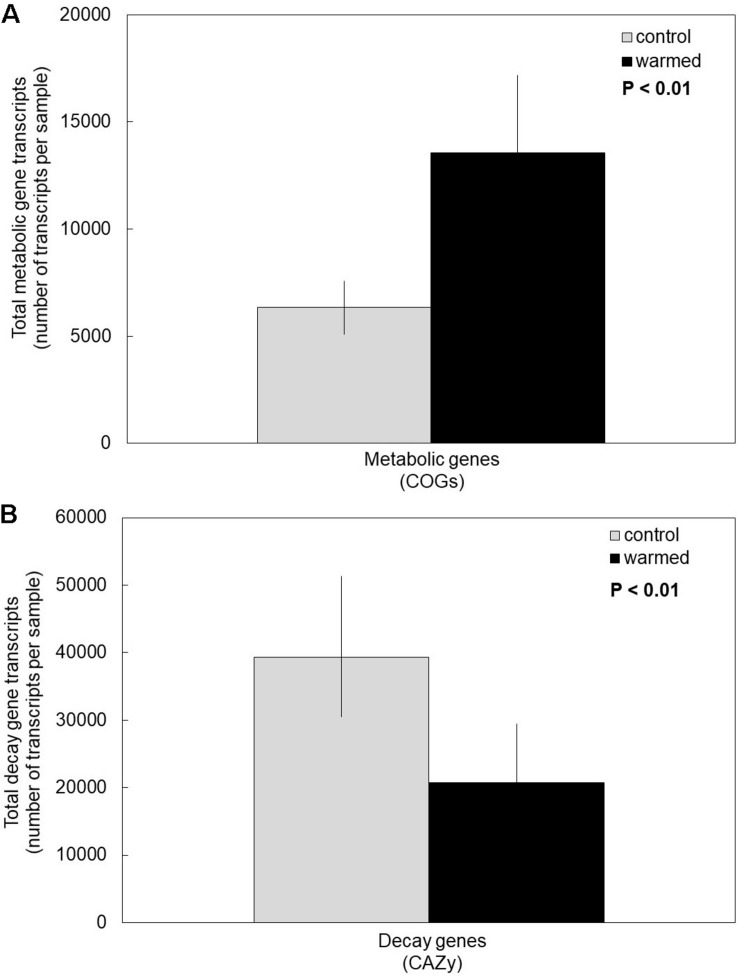
Cumulative average of transcripts in controls compared to the warming treatment for **(A)** metabolic clusters of orthologous groups (COG) and **(B)** carbohydrate-active enzyme classes (CAZy). Fungi in the warming treatment had on average significantly more transcripts of COG genes and significantly fewer transcripts of CAZy genes compared to controls. Bars are cumulative averages of four plots (*n* = 4) ± 1SE, *P* < 0.01 for treatment. Transcript counts of each sample and each COG subcategory and CAZy category are reported in Supplementary Tables S1, S2, respectively.

Under the warming treatment (which includes drying), fungi invested more in the transcription of cell metabolic maintenance genes compared to controls (Figure 1A). We accepted our first hypothesis, because the overall transcription of COG metabolic genes was significantly higher in the warmed treatment compared to control plots (Supplementary Table S1, *P* < 0.01). Moreover, there was no significant interaction between COG metabolic subcategory and warming treatment, indicating that COG categories responded similarly to the warming treatment (*P* = 0.97). Regardless of treatment, certain COG subcategories (e.g., energy production and conversion, amino acid transport and metabolism) were transcribed significantly more than others, as suggested by a significant main effect of COG subcategory (*P* < 0.01).

Conversely, under the warming treatment, fungi invested less in the transcription of genes for enzymes catabolizing glycosidic bonds (CAZy genes) compared to control temperatures (Figure 1B). Accordingly, our second hypothesis was supported (*P* < 0.01). The various CAZy classes responded similarly to the warming treatment, based on a non-significant treatment by CAZy class interaction (Supplementary Table S2, *P* = 0.36). Across treatments, glycoside hydrolases was the most transcribed CAZy class, and PL was the least (main effect of CAZy class: *P* = 0.02).

**FIGURE 2:**
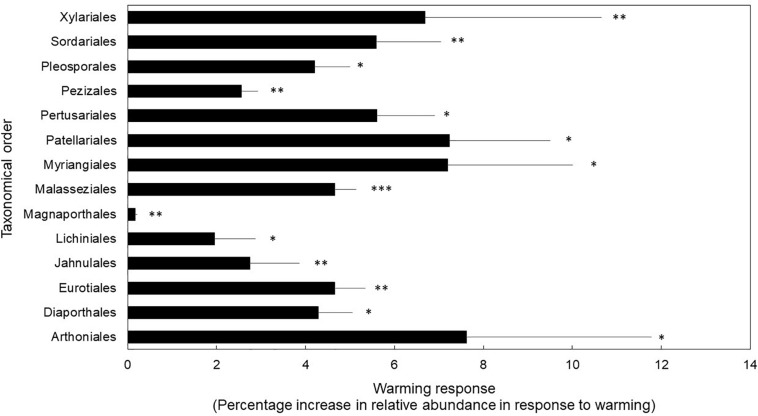
Fungal orders that displayed significantly different warming treatment response as percentage increase in relative abundance in response to warming. Bars are means ± 1SE of *n* = 4. ^∗^*P* < 0.05, ^∗∗^*P* < 0.01, ^∗∗∗^*P* < 0.001. Transcript counts and relative abundance of all orders in this study are reported in Supplementary Table S3.

Finally, fungal orders that house taxa with stress-tolerant traits (e.g., melanized cell walls and spores, desiccation-tolerance) were favored under the warming treatment (Figure 2). We found support for our third hypothesis because taxonomical orders with higher relative abundance in the warmed treatment compared to control plots are known for their stress-tolerance traits (*P* < 0.01 for treatment; and *P* < 0.01 for taxonomical orders). We found a significant interaction between treatment and taxonomical orders (*P* = 0.05). *Post hoc t*-test revealed that orders known to house lichenized, corticioid, melanized, high-sporulating, endophytic, pathogenic, and xerotolerant fungi were significantly more abundant in the warmed treatment compared to controls at *P* ≤ 0.05 (Supplementary Table S3). Specifically, the orders Arthoniales, Lichinales, and Pertusariales house lichenized fungi (DePriest, 2004; Schmitt et al., 2005; Lücking et al., 2017). The Diaporthales, Magnaporthales, and Malasseziales include mostly opportunistic pathogenic species (Cafarchia et al., 2007; Roy and Mulder, 2014; Zhang et al., 2016; Senanayake et al., 2017). The Eurotiales house high-sporulating, stress-tolerant mold species (Dijksterhuis et al., 2018). The Jahnulales, Myrangiales, Patellariales, and Pleosporales have species known to tolerate stress due to melanized cell walls (Ruibal et al., 2009; Schoch et al., 2009). The Pezizales and Sordariales house taxa that can thrive under stressful conditions (e.g., dry and hot post-fire conditions) (Egger, 1986; Grishkan et al., 2003). Finally, the Xylariales house many endophytic, stress-tolerant, melanized taxa (Yu et al., 2015; Soares et al., 2016).

## Discussion

In our study, we found that experimental warming treatments (which include drying) elicits an ecological tradeoff in fungal resource allocation which favors cell metabolic maintenance over decay (Figure 1). We also found that warming and drying causes an increase in abundance of fungal orders with stress-tolerant traits (Figure 2), which may be drivers of the observed ecological tradeoff.

Previous studies have shown that cell maintenance costs increase with temperature (Joergensen et al., 1990), causing higher energetic demand to maintain cellular function (Steinweg et al., 2013). This increase in energetic demand results in increased death rates of microbes (Alvarez et al., 1995; Curtin et al., 2012). Consequently, increased temperature that leads to microbial death or resource allocation tradeoffs can potentially shape fungal communities and ecosystem-scale C processes. In our study, fungi under warming and drying invested resources in cell metabolic maintenance potentially to ensure their survival under global climate change.

Other studies have also documented microbial ecological tradeoffs. Treseder et al. (2011) found a tradeoff between recalcitrant C users and nitrogen availability; recalcitrant C users were more prevalent under environments high in nitrates and low in organic nitrogen. When two processes are energetically expensive, an ecological tradeoff will favor acquisition of one trait that favors survival at the expense of losing another one. Similarly, Romero-Olivares et al. (2015) found that the model fungus *N. discreta* adapted to elevated temperatures by producing more spores at the expense of biomass. Because producing spores is energetically expensive, *N. discreta* could not afford investing in both, spore and biomass production. Thus, investing in resources which will secure reproduction was an effective survival strategy.

Treseder et al. (2016) studied community shifts in our field experiment by high-throughput sequencing of soil DNA. In the current study, we used a highly sensitive sequencing method based on soil RNA and found similar results; the Eurotiales, Pezizales, and Sordariales increased under warming and drying. Most of the orders we found at higher abundance under warming were taxa known to house lichenized, free-living, melanized, stress-tolerant, endophytic, and pathogenic fungi. Although some taxa displayed decreases in abundance in response to warming and drying, none of these were significantly different (e.g., Morteriellales, Cystofilobasidiales) (Supplementary Table S3). Interestingly, in our dataset, the Mortierellales, Mucorales, and other high-sporulating taxa were the most abundant taxa in the warming treatment and controls alike (Supplementary Table S3). Indeed, in culture isolation efforts carried out in these same soils by AL Romero-Olivares, *Umbelopsis* spp. *Ambomucor* spp., and *Penicillium* spp.—all high-sporulating taxa—were the most commonly isolated species in each treatment (data not published). This could indicate that the orders that were equally abundant in control and warming treatments were the result of spore banks in soils, which can be metabolically active (Novodvorska et al., 2016). Surprisingly, Basidiomycete fungi, known to play a key role in decomposition, were not significantly affected by the warming treatment. Specifically, no Basidiomycete orders decreased significantly under the warming treatment. In fact, the only Basidiomycete order that responded significantly to the warming treatment was the Malasseziales, which are known to house pathogenic yeasts (Figure 2 and Supplementary Table S3). Alternatively, Ascomycete orders were significantly affected by the warming treatment. Similar trends have been reported under long-term chronic nitrogen fertilization (Hesse et al., 2015).

Our findings are in line with mass loss litterbag experiments done in our field site. Here, we reported that resource allocation toward decomposition was lower in the warming treatment compared to controls. Romero-Olivares et al. (2017a) reported that mass loss and extracellular enzyme activity was lower in the warming treatment compared to controls. In contrast, they noted that the proportional loss of recalcitrant C in the warming treatment was greater than in controls. Differential expression analysis showed that AA genes, which are involved in the decomposition of recalcitrant C, tended to be transcribed at (non-significantly) higher rates in the warming treatment compared to controls (Supplementary Table S2 and Supplementary Figure S1). In contrast, CE and GH genes tended to be transcribed (significantly) more in controls than in the warming treatment. While the area of soil metatranscriptomes continues to develop, we suggest accompanying metatranscriptomes with field observations to draw ecologically relevant conclusions.

As the area of soil metatranscriptomics develops, sampling efforts should likewise increase. This goal is important, since fungi communities residing in different ecosystems might vary in their responses to warming and drying (Wallenstein and Hall, 2012). For example, deserts, savannas, tropical forests, and other particularly warm ecosystems may already be dominated by stress tolerant taxa. In those cases, shifts between cell maintenance and decay might not be as strongly evident. At the moment, sampling efforts are hampered by insufficient random access memory (i.e., RAM), even for high-performance computer clusters. The amount of sequencing data in metatranscriptomes is massive (i.e., ∼0.5 TB of sequencing data per sample), and analysis platforms require high RAM in order to successfully process the data. As such, we limited our sample collections to one sampling time in the middle of the summer. Thus, our results represent a snapshot of the time and conditions of that particular day and time in this specific ecosystem and generalization is impossible. In the future, transcript abundance should be analyzed throughout a day, week, months, and years. This is especially important because similar studies examining temporal changes of microbial activity have shown that fungal transcription in soil differs among seasons (Žifčáková et al., 2016). In fact, CAZy GH and AA genes are transcribed at higher rates in summer compared to winter (Žifčáková et al., 2017).

Moreover, preservation methods for RNA in soil samples are also critical. Flash freezing in liquid nitrogen may be the best preservation method for RNA in soil samples (e.g., Žifčáková et al., 2016, 2017). However, this approach was not feasible in our remote field site. Instead, we used LifeGuard^TM^ Soil Preservation Solution. We caution that the preservation liquid may not reach every single cell in the soil fast enough to capture all RNA. In fact, the effect of preservation methods in forest soils has not been thoroughly compared yet (Tatangelo et al., 2014); when flash freezing in liquid nitrogen is not a feasible option, LifeGuard^TM^ Soil Preservation Solution seems to be the most widely used option (e.g., Carvalhais et al., 2012; Che et al., 2017; Jurburg et al., 2017; Meredith et al., 2018; Rampadarath et al., 2018; Thompson et al., 2018). In addition, pipelines for processing and analyzing metatranscriptomes in the context of fungi and soil C processes are still developing (Baldrian and López-Mondéjar, 2014). Vast efforts are underway to develop pipelines for *de novo* metatranscriptomes (e.g., Haas et al., 2013) and improve fungal annotations (Grigoriev et al., 2014). Our work is the result of the existing information available in databases and pipelines for processing and analyzing transcriptomes without reference genomes.

In conclusion, our data suggest that under long-term warming and drying, fungal communities allocate resources toward cell metabolic maintenance at the expense of decomposition. In addition, fungi with stress-tolerance traits, such as melanized, xerotolerant, and lichenized fungi, seem to prevail under warming and drying. The metatranscriptome approach allowed us to pin-point, at the gene level, the mechanisms behind ecosystem-scale observations on soil C processes. We present a pipeline that paired with field observations, provides insight into the genetic mechanisms behind resource allocation efforts of fungi under long-term experimental warming and drying. It can help in predicting the fate of soil C under global climate change. In our study, soil metatranscriptomes provide a mechanistic snapshot of C-related ecological processes happening under climate change and offer insight into microbial resource allocation which are in-line with field observations.

## Data Availability

The datasets generated for this study can be found in the JGI project IDs 1107-496, -499, -504, -507, -509, -514, -519, and -520 and MG-RAST metagenomics analysis server with the following IDs: mgm4761695.3, mgm4762031.3, mgm4762032.3, mgm4762033.3, mgm4762030.3, mgm4762034.3, mgm4762035.3, and mgm4762036.3.

## Author Contributions

AR-O conceived and designed the study, performed the research, analyzed the data, and wrote the manuscript. GM-C provided support with the data processing and helped to revise the manuscript. AL-L helped in the data processing and revising the manuscript. KT conceived and designed the study, and helped to revise the manuscript.

## Conflict of Interest Statement

The authors declare that the research was conducted in the absence of any commercial or financial relationships that could be construed as a potential conflict of interest.

## Footnotes

1 https://github.com/TransDecoder/TransDecoder

2 https://github.com/Trinotate/Trinotate.github.io/wiki

## Supplementary Material

FIGURE S1 Heatmap with significantly different Log^2^ differential expression at *P* ≤ 0.01 in controls and the warming treatment. Red shows down-regulated genes (−10 to −1), green shows up-regulated genes (1 to 10), and yellow down- or up-regulated genes (−0.99 to 0.99). Samples are organized by control and adjacent warming treatment plot. Control samples are C1, C2, C3, C4, and warming treatment samples are W1, W2, W3, W4 (*n* = 4). Y-axes shows CAZy class: AA, Auxiliary Activities; CBM, Carbohydrate Binding Modules; CE, Carbohydrate Esterases; GH, Glycoside Hydrolases; PL, Polysaccharide Lyases. *Post hoc p*-values for each category are shown underneath Y-axes categories.

TABLE S1 Total transcripts of predicted metabolic clusters of orthologous groups (COG) genes in control and warmed samples (*n* = 4).

TABLE S2 Total transcripts of predicted carbohydrate-active enzyme classes (CAZy) genes in control and warmed samples (*n* = 4).

TABLE S3 Total transcripts and relative abundance of fungal orders in control and warmed samples (*n* = 4).

## References

[B1] Albright M. B. N. Johansen R. Lopez D. Gallegos-Graves L. V. Steven B. Kuske C. R. (2018). Short-term transcriptional response of microbial communities to nitrogen fertilization in a pine forest soil. *Appl. Environ. Microbiol.* 84:e00598-18. 10.1128/AEM.00598-18 29802185 PMC6052259

[B2] Allison S. D. (2014). Modeling adaptation of carbon use efficiency in microbial communities. *Front. Microbiol.* 5:571 10.3389/fmicb.2014.00571 PMC4211550 25389423

[B3] Allison S. D. Romero-Olivares A. L. Lu L. Taylor J. Treseder K. K. (2018a). Temperature acclimation and adaptation of enzyme physiology in *Neurospora discreta*. *Fungal Ecol.* 35 78–86. 10.1016/j.funeco.2018.07.005

[B4] Allison S. D. Romero-Olivares A. L. Lu Y. Taylor J. W. Treseder K. K. (2018b). Temperature sensitivities of extracellular enzyme Vmax and Km across thermal environments. *Glob. Chang. Biol.* 24 2884–2897. 29322601 10.1111/gcb.14045

[B5] Allison S. D. Treseder K. K. (2008). Warming and drying suppress microbial activity and carbon cycling in boreal forest soils. *Glob. Chang. Biol.* 14 2898–2909. 10.1111/j.1365-2486.2008.01716.x

[B6] Allison S. D. Treseder K. K. (2011). Climate change feedbacks to microbial decomposition in boreal soils. *Fungal Ecol.* 4 362–374. 10.1016/j.funeco.2011.01.003

[B7] Alvarez R. Santanatoglia O. J. Garcia R. (1995). Effect of temperature on soil microbial biomass and its metabolic quotient in situ under different tillage systems. *Biol. Fertil. Soils* 19 227–230. 10.1007/bf00336164

[B8] Andrews S. (2010). *FastQC**: A Quality Control Tool for High Throughput Sequence Data.* Available at: https://www.bioinformatics.babraham.ac.uk/projects/fastqc/ (accessed November 2016).

[B9] Azua-Bustos A. Urrejola C. Vicuña R. (2012). Life at the dry edge: microorganisms of the atacama desert. *FEBS Lett.* 586 2939–2945. 10.1016/j.febslet.2012.07.025 22819826

[B10] Baldrian P. (2017). Forest microbiome: diversity, complexity and dynamics. *FEMS Microbiol. Rev.* 41 109–130. 10.1093/femsre/fuw040 27856492

[B11] Baldrian P. Kolařík M. Štursová M. Kopecký J. Valášková V. Větrovský T. (2012). Active and total microbial communities in forest soil are largely different and highly stratified during decomposition. *ISME J.* 6 248–258. 10.1038/ismej.2011.95 21776033 PMC3260513

[B12] Baldrian P. López-Mondéjar R. (2014). Microbial genomics, transcriptomics and proteomics: new discoveries in decomposition research using complementary methods. *Appl. Microbiol. Biotechnol.* 98 1531–1537. 10.1007/s00253-013-5457-x 24384749

[B13] Bates D. Mächler M. Bolker B. M. Walker S. C. (2015). Fitting linear mixed-effects models using lme4. *J. Stat. Softw.* 67:54759 10.18637/jss.v067.i01

[B14] Belozerskaya T. Aslanidi K. Ivanova A. Gessler N. Egorova A. Karpenko Y. (2010). “Characteristics of extremophylic fungi from chernobyl nuclear power,” in *Current Research, Technology and Education Topics in Applied Microbiology and Microbial Biotechnology*, ed. Mendez-Vilas A. (Bajadoz: Formatex), 88–94.

[B15] Bennett A. F. Lenski R. E. (2007). An experimental test of evolutionary trade-offs during temperature adaptation. *Proc. Natl. Acad. Sci. U.S.A.* 104(Suppl. 1), 8649–8654. 10.1073/pnas.0702117104 17494741 PMC1876442

[B16] Berlemont R. (2017). Distribution and diversity of enzymes for polysaccharide degradation in fungi. *Sci. Rep.* 7 1–11. 10.1038/s41598-017-00258-w 28302998 PMC5428031

[B17] Bolger A. M. Lohse M. Usadel B. (2014). Trimmomatic: a flexible trimmer for Illumina sequence data. *Bioinformatics* 30 2114–2120. 10.1093/bioinformatics/btu170 24695404 PMC4103590

[B18] Bryant D. M. Johnson K. DiTommaso T. Tickle T. Couger M. B. Payzin-Dogru D. (2017). A tissue-mapped axolotl de novo transcriptome enables identification of limb regeneration factors. *Cell Rep.* 18 762–776. 10.1016/j.celrep.2016.12.063 28099853 PMC5419050

[B19] Cafarchia C. Otranto D. Campbell B. E. Latrofa M. S. Guillot J. Gasser R. B. (2007). Multilocus mutation scanning for the analysis of genetic variation within *Malassezia* (*Basidiomycota*: Malasseziales). *Electrophoresis* 28 1176–1180. 10.1002/elps.200600624 17514784

[B20] Carvalhais L. C. Dennis P. G. Tyson G. W. Schenk P. M. (2012). Application of metatranscriptomics to soil environments. *J. Microbiol. Methods* 91 246–251. 10.1016/j.mimet.2012.08.011 22963791

[B21] Casadevall A. Cordero R. J. B. Bryan R. Nosanchuk J. Dadachova E. (2017). Melanin, radiation, and energy transduction in fungi. *Microbiol. Spectr.* 5 1–6. 10.1128/microbiolspec.FUNK-0037-2016 28256187 PMC11687467

[B22] Che R. Wang F. Wang W. Zhang J. Zhao X. Rui Y. (2017). Increase in ammonia-oxidizing microbe abundance during degradation of alpine meadows may lead to greater soil nitrogen loss. *Biogeochemistry* 136 341–352. 10.1007/s10533-017-0399-395

[B23] Chen I.-M. A. Markowitz V. M. Chu K. Palaniappan K. Szeto E. Pillay M. (2016). IMG/M: integrated genome and metagenome comparative data analysis system. *Nucleic Acids Res.* 45 D507–D516. 10.1093/nar/gkw929 27738135 PMC5210632

[B24] Crowther T. W. Maynard D. S. Crowther T. R. Peccia J. Smith J. R. Bradford M. A. (2014). Untangling the fungal niche: the trait-based approach. *Front. Microbiol.* 5:597. 10.3389/fmicb.2014.00579 25400630 PMC4215788

[B25] Curtin D. Beare M. H. Hernandez-Ramirez G. (2012). Temperature and moisture effects on microbial biomass and soil organic matter mineralization. *Soil Sci. Soc. Am. J.* 76 2055–2067. 10.2136/sssaj2012.0011

[B26] DePriest P. T. (2004). Early molecular investigations of lichen-forming symbionts: 1986–2001. *Annu. Rev. Microbiol.* 58 273–301. 10.1146/annurev.micro.58.030603.123730 15487939

[B27] Dijksterhuis J. Meijer M. van Doorn T. Samson R. Rico-Munoz E. (2018). Inactivation of stress-resistant ascospores of eurotiales by industrial sanitizers. *Int. J. Food Microbiol.* 285 27–33. 10.1016/j.ijfoodmicro.2018.06.018 30015260

[B28] Egger K. N. (1986). Substrate hydrolysis patterns of post-fire ascomycetes (*Pezizales*). *Mycologia* 78 771–780. 10.1080/00275514.1986.12025321

[B29] Farmer I. S. Jones C. W. (1976). The effect of temperature on the molar growth yield and maintenance requirement of *Escherichia coli* W during aerobic growth in continuous culture. *FEBS* 67 359–363. 10.1016/0014-5793(76)80564-9 786728

[B30] Fernandez C. W. Koide R. T. (2013). The function of melanin in the ectomycorrhizal fungus *Cenococcum geophilum* under water stress. *Fungal Ecol.* 6 479–486. 10.1016/j.funeco.2013.08.004

[B31] Frank S. A. (2010). The trade-off between rate and yield in the design of microbial metabolism. *J. Evol. Biol.* 23 609–613. 10.1111/j.1420-9101.2010.01930.x 20487133

[B32] Gasch A. P. Spellman P. T. Kao C. M. Carmel-Harel O. Eisen M. B. Storz G. (2000). Genomic expression programs in the response of yeast cells to environmental changes. *Mol. Biol. Cell* 11 4241–4257. 10.1091/mbc.11.12.4241 11102521 PMC15070

[B33] Grigoriev I. V. Nikitin R. Haridas S. Kuo A. Ohm R. Otillar R. (2014). MycoCosm portal: gearing up for 1000 fungal genomes. *Nucleic Acids Res.* 42 699–704. 10.1093/nar/gkt1183 24297253 PMC3965089

[B34] Grishkan I. Korol A. B. Nevo E. Wasser S. P. (2003). Ecological stress and sex evolution in soil microfungi. *Proc. R. Soc. B Biol. Sci.* 270 13–18. 10.1098/rspb.2002.2194 12590766 PMC1691208

[B35] Gunde-Cimerman N. Sonjak S. Zalar P. Frisvad J. C. Diderichsen B. Plemenitaš A. (2003). Extremophilic fungi in arctic ice: a relationship between adaptation to low temperature and water activity. *Phys. Chem. Earth* 28 1273–1278. 10.1016/j.pce.2003.08.056

[B36] Haas B. J. Papanicolaou A. Yassour M. Grabherr M. Blood P. D. Bowden J. (2013). De novo transcript sequence reconstruction from RNA-seq using the trinity platform for reference generation and analysis. *Nat. Protoc.* 8 1494–1512. 10.1038/nprot.2013.084 23845962 PMC3875132

[B37] Hall E. K. Cotner J. B. (2007). Interactive effect of temperature and resources on carbon cycling by freshwater bacterioplankton communities. *Aquat. Microb. Ecol.* 49 35–45. 10.3354/ame01124

[B38] Harutyunyan S. Muggia L. Grube M. (2008). Black fungi in lichens from seasonally arid habitats. *Stud. Mycol.* 61 83–90. 10.3114/sim.2008.61.08 19287530 PMC2610299

[B39] Hesse C. N. Mueller R. C. Vuyisich M. Gallegos-Graves L. V. Gleasner C. D. Zak D. R. (2015). Forest floor community metatranscriptomes identify fungal and bacterial responses to N deposition in two maple forests. *Front. Microbiol.* 6:337. 10.3389/fmicb.2015.00337 25954263 PMC4407611

[B40] Joergensen R. G. Brookes P. C. Jenkinson D. S. (1990). Survival of the soil microbial biomass at elevated temperatures. *Soil Biol. Biochem.* 22 1129–1136. 10.1016/0038-0717(90)90039-3

[B41] Jurburg S. D. Nunes I. Stegen J. C. Le Roux X. Priemé A. Sørensen S. J. (2017). Autogenic succession and deterministic recovery following disturbance in soil bacterial communities. *Sci. Rep.* 7 1–11. 10.1038/srep45691 28383027 PMC5382530

[B42] Kopylova E. Noé L. Touzet H. (2012). SortMeRNA: fast and accurate filtering of ribosomal RNAs in metatranscriptomic data. *Bioinformatics* 28 3211–3217. 10.1093/bioinformatics/bts611 23071270

[B43] Kuznetsova A. Brockhoff P. B. Christensen R. H. B. (2017). lmertest package: tests in linear mixed effects models. *J. Stat. Softw.* 82:15084 10.18637/jss.v082.i13

[B44] Langmead B. Salzberg S. L. (2013). Fast gapped-read alignment with bowtie2. *Nat. Methods* 9 357–359. 10.1038/nmeth.1923.Fast 22388286 PMC3322381

[B45] Li H. Handsaker B. Wysoker A. Fennell T. Ruan J. Homer N. (2009). The sequence alignment/map format and SAMtools. *Bioinformatics* 25 2078–2079. 10.1093/bioinformatics/btp352 19505943 PMC2723002

[B46] Lombard V. Golaconda Ramulu H. Drula E. Coutinho P. M. Henrissat B. (2014). The carbohydrate-active enzymes database (CAZy) in 2013. *Nucleic Acids Res.* 42 490–495. 10.1093/nar/gkt1178 24270786 PMC3965031

[B47] Lücking R. Hodkinson B. P. Leavitt S. D. (2017). The 2016 classification of lichenized fungi in the *Ascomycota* and basidiomycota – approaching one thousand genera. *Bryologist* 119 361–416. 10.1639/0007-2745-119.4.361

[B48] Mainzer S. E. Hempfling W. P. (1976). Effects of growth temperature on yield and maintenance during glucose-limited continuous culture of *Escherichia coli*. *J. Bacteriol.* 126 251–256. 770423 10.1128/jb.126.1.251-256.1976 PMC233282

[B49] Markowitz V. M. Chen I.-M. A. Palaniappan K. Chu K. Szeto E. Grechkin Y. (2005). *IMG: the Integrated Microbial Genomes Database and Comparative Analysis System.* Available at: https://img.jgi.doe.gov/ (accessed May 2017). 10.1093/nar/gkr1044 PMC3245086 22194640

[B50] Melillo J. M. Frey S. D. Deangelis K. M. Werner W. J. Bernard M. J. Bowles F. P. (2017). Long-term pattern and magnitude of soil carbon feedback to the climate system in a warming world. *Science* 358 101–105. 10.1126/science.aan2874 28983050

[B51] Meredith L. K. Boye K. Youngerman C. Whelan M. Ogée J. Sauze J. (2018). Coupled biological and abiotic mechanisms driving carbonyl sulfide production in soils. *Soil Syst.* 2:37 10.3390/soilsystems2030037

[B52] Meyer F. Paarman D. D’Souza M. Olson R. Glass E. Kubal M. (2008). The metagenomics RAST server — a public resource for the automatic phylogenetic and functional analysis of metagenomes. *BMC Bioinformatics* 9:386. 10.1186/1471-2105-9-386 18803844 PMC2563014

[B53] Mittermeier V. K. Schmitt N. Volk L. P. M. Suárez J. P. Beck A. Eisenreich W. (2015). Metabolic profiling of alpine and ecuadorian lichens. *Molecules* 20 18047–18065. 10.3390/molecules201018047 26437395 PMC6332210

[B54] Morrison E. W. Pringle A. Diepen L. T. A. Van Grandy A. S. Melillo J. M. Frey S. D. (2019). Warming alters fungal communities and litter chemistry with implications for soil carbon stocks. *Soil Biol. Biochem.* 132 120–130. 10.1016/j.soilbio.2019.02.005

[B55] Nordberg H. Cantor M. Dusheyko S. Hua S. Poliakov A. Shabalov I. (2014). The genome portal of the department of energy joint genome institute: 2014 updates. *Nucleic Acids Res.* 42 26–31. 10.1093/nar/gkt1069 24225321 PMC3965075

[B56] Novodvorska M. Stratford M. Blythe M. J. Wilson R. Beniston R. G. Archer D. B. (2016). Metabolic activity in dormant conidia of *Aspergillus niger* and developmental changes during conidial outgrowth. *Fungal Genet. Biol.* 94 23–31. 10.1016/j.fgb.2016.07.002 27378203 PMC4981222

[B57] Pold G. Melillo J. M. DeAngelis K. M. (2015). Two decades of warming increases diversity of a potentially lignolytic bacterial community. *Front. Microbiol.* 6:480. 10.3389/fmicb.2015.00480 26042112 PMC4438230

[B58] Quast C. Pruesse E. Yilmaz P. Gerken J. Schweer T. Yarza P. (2013). The SILVA ribosomal RNA gene database project: improved data processing and web-based tools. *Nucleic Acids Res.* 41 590–596. 10.1093/nar/gks1219 23193283 PMC3531112

[B59] R Core Team (2018). *R: a Language and Environment for Statistical Computing.* Available at: https://www.r-project.org (accessed December 2018).

[B60] Rampadarath S. Bandhoa K. Puchooa D. Jeewon R. Bal S. (2018). Metatranscriptomics analysis of mangroves habitats around Mauritius. *World J. Microbiol. Biotechnol.* 34 1–11. 10.1007/s11274-018-2442-2447 29611003

[B61] Randle-Boggis R. J. Helgason T. Sapp M. Ashton P. D. (2016). Evaluating techniques for metagenome annotation using simulated sequence data. *FEMS Microbiol. Ecol.* 92 1–15. 10.1093/femsec/fiw095 27162180 PMC4892715

[B62] Rodríguez-Verdugo A. Carrillo-Cisneros D. González-González A. Gaut B. S. Bennett A. F. (2014). Different tradeoffs result from alternate genetic adaptations to a common environment. *Proc. Natl. Acad. Sci. U.S.A.* 111 12121–12126. 10.1073/pnas.1406886111 25092325 PMC4143048

[B63] Romero-Olivares A. L. Allison S. D. Treseder K. K. (2017a). Decomposition of recalcitrant carbon under experimental warming in boreal forest. *PLoS One* 12:e0179674. 10.1371/journal.pone.0179674 28622366 PMC5473569

[B64] Romero-Olivares A. L. Allison S. D. Treseder K. K. (2017b). Soil microbes and their response to experimental warming over time: a meta-analysis of field studies. *Soil Biol. Biochem.* 107 32–40. 10.1016/j.soilbio.2016.12.026

[B65] Romero-Olivares A. L. Taylor J. W. Treseder K. K. (2015). *Neurospora discreta* as a model to assess adaptation of soil fungi to warming. *BMC Evol. Biol.* 15:198. 10.1186/s12862-015-0482-482 26377599 PMC4573461

[B66] Roy B. A. Mulder C. P. H. (2014). Pathogens, herbivores, and phenotypic plasticity of boreal *Vaccinium* vitis-idaea experiencing climate change. *Ecosphere* 5 1–19. 10.1890/ES13-00271.1

[B67] Ruibal C. Gueidan C. Selbmann L. Gorbushina A. A. Crous P. W. Groenewald J. Z. (2009). Phylogeny of rock-inhabiting fungi related to *Dothideomycetes*. *Stud. Mycol.* 64 123–133. 10.3114/sim.2009.64.06 20169026 PMC2816969

[B68] Schimel J. P. Balser T. C. Wallenstein M. D. (2007). Microbial stress-response physiology and its implications. *Ecology* 88 1386–1394. 10.1890/06-0219 17601131

[B69] Schmitt I. Martín M. P. Kautz S. Lumbsch H. T. (2005). Diversity of non-reducing polyketide synthase genes in the Pertusariales (lichenized *Ascomycota*): a phylogenetic perspective. *Phytochemistry* 66 1241–1253. 10.1016/j.phytochem.2005.04.014 15927215

[B70] Schoch C. L. Crous P. W. Groenewald J. Z. Boehm E. W. A. Burgess T. I. de Gruyter J. (2009). A class-wide phylogenetic assessment of *Dothideomycetes*. *Stud. Mycol.* 64 1–15. 10.3114/sim.2009.64.01 20169021 PMC2816964

[B71] Senanayake I. C. Crous P. W. Groenewald J. Z. Maharachchikumbura S. S. N. Jeewon R. Phillips A. J. L. (2017). Families of *Diaporthales* based on morphological and phylogenetic evidence. *Stud. Mycol.* 86 217–296. 10.1016/j.simyco.2017.07.003 28947840 PMC5603113

[B72] Soares M. A. Li H. Y. Kowalski K. P. Bergen M. Torres M. S. White J. F. (2016). Evaluation of the functional roles of fungal endophytes of *Phragmites australis* from high saline and low saline habitats. *Biol. Invasions* 18 2689–2702. 10.1007/s10530-016-1160-z

[B73] Solly E. F. Lindahl B. D. Dawes M. A. Peter M. Souza R. C. Rixen C. (2017). Experimental soil warming shifts the fungal community composition at the alpine treeline. *New Phytol.* 215 766–778. 10.1111/nph.14603 28543616

[B74] Steinweg M. J. Dukes J. S. Paul E. A. Wallenstein M. D. (2013). Microbial responses to multi-factor climate change: effects on soil enzymes. *Front. Microbiol.* 4:146. 10.3389/fmicb.2013.00146 23781218 PMC3678102

[B75] Tatangelo V. Franzetti A. Gandolfi I. Bestetti G. Ambrosini R. (2014). Effect of preservation method on the assessment of bacterial community structure in soil and water samples. *FEMS Microbiol. Lett.* 356 32–38. 10.1111/1574-6968.12475 24840085

[B76] Tatusov R. L. Galperin M. Y. Natale D. A. Koonin E. V. (2000). The COG database: a tool for genome-scale analysis of protein functions and evolution. *Nucleic Acids Res.* 28 33–36. 10.1093/nar/28.1.33 10592175 PMC102395

[B77] Thompson K. A. Deen B. Dunfield K. E. (2018). Impacts of surface-applied residues on N-cycling soil microbial communities in miscanthus and switchgrass cropping systems. *Appl. Soil Ecol.* 130 79–83. 10.1016/j.apsoil.2018.06.005

[B78] Treseder K. K. Kivlin S. N. Hawkes C. V. (2011). Evolutionary trade-offs among decomposers determine responses to nitrogen enrichment. *Ecol. Lett.* 14 933–938. 10.1111/j.1461-0248.2011.01650.x 21749597

[B79] Treseder K. K. Lennon J. T. (2015). Fungal traits that drive ecosystem dynamics on Land. *Microbiol. Mol. Biol. Rev.* 79 243–262. 10.1128/MMBR.00001-15 25971588 PMC4429240

[B80] Treseder K. K. Marusenko Y. Romero-Olivares A. L. Maltz M. R. (2016). Experimental warming alters potential function of the fungal community in boreal forest. *Glob. Chang. Biol.* 22 3395–3404. 10.1111/gcb.13238 26836961

[B81] Wallenstein M. D. Hall E. K. (2012). A trait-based framework for predicting when and where microbial adaptation to climate change will affect ecosystem functioning. *Biogeochemistry* 109 35–47. 10.1007/s10533-011-9641-9648

[B82] Yu X. Huo L. Liu H. Chen L. Wang Y. Zhu X. (2015). Melanin is required for the formation of the multi-cellular conidia in the endophytic fungus *Pestalotiopsis microspora*. *Microbiol. Res.* 179 1–11. 10.1016/j.micres.2015.06.004 26411889

[B83] Zhang N. Luo J. Rossman A. Y. Aoki T. Chuma I. Crous P. W. (2016). Generic names in *Magnaporthales*. *IMA Fungus* 7 155–159. 10.5598/imafungus.2016.07.01.09 27433445 PMC4941683

[B84] Žifčáková L. Větrovský T. Howe A. Baldrian P. (2016). Microbial activity in forest soil reflects the changes in ecosystem properties between summer and winter. *Environ. Microbiol.* 18 288–301. 10.1111/1462-2920.13026 26286355

[B85] Žifčáková L. Větrovský T. Lombard V. Henrissat B. Howe A. Baldrian P. (2017). Feed in summer, rest in winter: microbial carbon utilization in forest topsoil. *Microbiome* 5:122. 10.1186/s40168-017-0340-340 28923122 PMC5604414

