# Supplementary figures and images for: Soil Metatranscriptomes Under Long-Term Experimental Warming and Drying: Fungi Allocate Resources to Cell Metabolic Maintenance Rather Than Decay
Source: Front Microbiol. 2019 Aug 20;10:1914. doi: 10.3389/fmicb.2019.01914 (PMC6736569; doi:10.3389/fmicb.2019.01914)

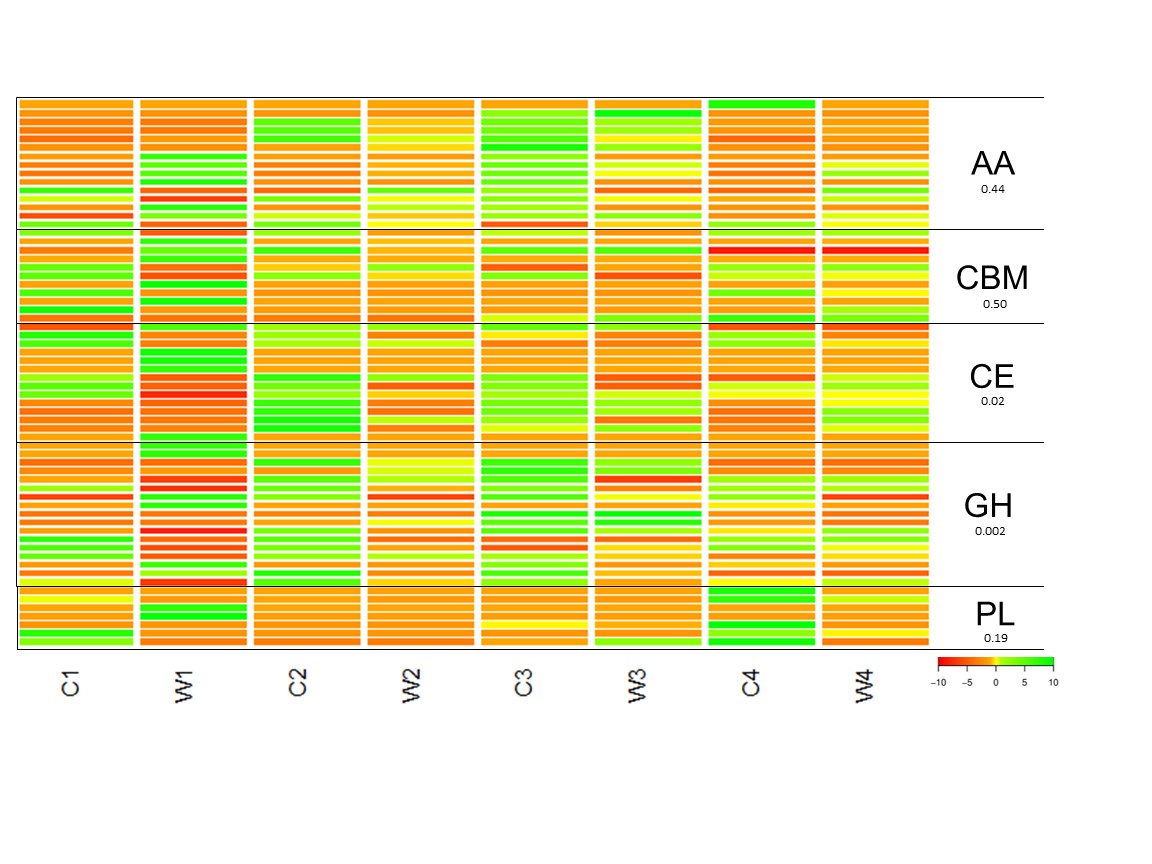

Supplement: FIGURE S1 — Heatmap with significantly different Log2 differential expression at P ≤ 0.01 in controls and the warming treatment. Red shows down-regulated genes (−10 to −1), green shows up-regulated genes (1 to 10), and yellow down- or up-regulated genes (−0.99 to 0.99). Samples are organized by control and adjacent warming treatment plot. Control samples are C1, C2, C3, C4, and warming treatment samples are W1, W2, W3, W4 (n = 4). Y-axes shows CAZy class: AA, Auxiliary Activities; CBM, Carbohydrate Binding Modules; CE, Carbohydrate Esterases; GH, Glycoside Hydrolases; PL, Polysaccharide Lyases. Post hoc p-values for each category are shown underneath Y-axes categories. [file Image_1.JPEG]
